# Profiling of Olfactory Receptor Gene Expression in Whole Human Olfactory Mucosa

**DOI:** 10.1371/journal.pone.0096333

**Published:** 2014-05-06

**Authors:** Christophe Verbeurgt, Françoise Wilkin, Maxime Tarabichi, Françoise Gregoire, Jacques E. Dumont, Pierre Chatelain

**Affiliations:** 1 ChemCom S.A., Brussels, Belgium; 2 Department of Otorhinolaryngology, Erasme University Hospital, Brussels, Belgium; 3 Institute of Interdisciplinary Research in human and molecular Biology, Free University of Brussels, Brussels, Belgium; 4 Laboratory of Pathophysiological and Nutritional Biochemistry, Department of Biochemistry, Free University of Brussels, Brussels, Belgium; Plant and Food Research, New Zealand

## Abstract

Olfactory perception is mediated by a large array of olfactory receptor genes. The human genome contains 851 olfactory receptor gene loci. More than 50% of the loci are annotated as nonfunctional due to frame-disrupting mutations. Furthermore haplotypic missense alleles can be nonfunctional resulting from substitution of key amino acids governing protein folding or interactions with signal transduction components. Beyond their role in odor recognition, functional olfactory receptors are also required for a proper targeting of olfactory neuron axons to their corresponding glomeruli in the olfactory bulb. Therefore, we anticipate that profiling of olfactory receptor gene expression in whole human olfactory mucosa and analysis in the human population of their expression should provide an opportunity to select the frequently expressed and potentially functional olfactory receptors in view of a systematic deorphanization. To address this issue, we designed a TaqMan Low Density Array (Applied Biosystems), containing probes for 356 predicted human olfactory receptor loci to investigate their expression in whole human olfactory mucosa tissues from 26 individuals (13 women, 13 men; aged from 39 to 81 years, with an average of 67±11 years for women and 63±12 years for men). Total RNA isolation, DNase treatment, RNA integrity evaluation and reverse transcription were performed for these 26 samples. Then 384 targeted genes (including endogenous control genes and reference genes specifically expressed in olfactory epithelium for normalization purpose) were analyzed using the same real-time reverse transcription PCR platform. On average, the expression of 273 human olfactory receptor genes was observed in the 26 selected whole human olfactory mucosa analyzed, of which 90 were expressed in all 26 individuals. Most of the olfactory receptors deorphanized to date on the basis of sensitivity to known odorant molecules, which are described in the literature, were found in the expressed olfactory receptors gene set.

## Introduction

Analysis of published mammalian genomes indicates that olfactory receptor (OR) genes constitute by far the largest gene family. Initially, Buck and Axel identified this extremely large multigene family based on the observation that OR genes were expressed in olfactory epithelium of rat [1]. Later, other members of this family were identified by sequence homology with the first set of OR genes [2]–[4]. Currently it is accepted that the human genome contains 851 OR loci. More than 50% of the loci are annotated as nonfunctional due to frame-disrupting mutations, leaving approximately 400 potentially functional OR genes.

In spite of this rather accurate genomic characterization, very little is known of the functional and integrative mechanisms of human olfactory receptor in odorant perception. To date, the responses of only 48 human ORs with one or more odorant molecules have been reported [5]–[20] and less than ten of these receptors have been reliably associated with olfactory perception of an odorant stimuli [8]–[10], [13], [14].

In the quest to develop industrial applications based on the use of human odorant receptors, ChemCom is committed to the systematic identification of ligands for these chemoreceptors. To fulfill this ambitious deorphanization project, considering the huge number of anticipated functional OR genes, it is mandatory to obtain clues about the involvement of the targeted ORs in the olfactory perception. Moreover, the expression of several predicted OR genes has been detected in non-olfactory tissues, suggesting that a subset of predicted OR genes could have functions unrelated to olfaction. Indeed, expression of OR transcripts has been described in various tissues, including testis and spermatozoa [19], [21]–[26], prostate [27]–[30], enterochromaffin cells [6], pulmonary neuroendocrine cells [31], brain [32]–[35], tongue [36]–[38], erythroid cells [39], placenta [40], breast [41] and kidney [42]. In addition, systematic expression profiling of ORs in non-olfactory tissues using EST data, microarray or deep sequencing analysis [43]–[45] have shown that a large number of putative human OR genes are expressed in these tissues. The analysis of the entire olfactory subtranscriptome in a variety of different human tissues provides a list of several OR genes that are highly expressed in non-olfactory tissues [44]. At least some of these ORs could play a role in spermatozoa chemotactism [19], in muscle regeneration [46] or in blood pressure regulation [47]. Although, it cannot be excluded that OR may present double olfactory and non-olfactory functions; it remains possible that some members of the reported odorant receptors family could be solely non-olfactory G protein-coupled receptors.

Another issue pertaining to ORs deorphanization results from the significant allelic variation observed for human ORs. A recent data mining of the sequence repository of the 1000 Genomes Project, has estimated that the number of variants per OR locus is on average about ten. However, some variants may be nonfunctional missense haplotypic alleles [48]. Furthermore, as it has been demonstrated in mice that functional olfactory receptors are required for proper targeting of olfactory neuron axons to their corresponding glomeruli in the olfactory bulb [49], one may suppose that alleles of OR genes predominantly expressed in the olfactory epithelium correspond to functional haplotypes.

Taken together, a study of OR gene expression in the whole human olfactory mucosa (WHOM) provides an opportunity to define ORs specifically involved in olfaction, allowing choosing frequently expressed and potentially functional ORs for deorphanization campaigns. OR gene expression in WHOM has been seldomly studied, probably due to the difficult access to human material. Two publications have reported the characterization of the expression of the human OR gene family in 3 individuals only using DNA microarray and only in one individual using deep sequencing [45], [50]. Therefore, we designed an innovative approach based on a TaqMan Low Density Array (TLDA) containing probes for 356 predicted OR loci to investigate more thoroughly the OR gene expression profile in human olfactory mucosa. Real-time reverse transcription PCR (qRT-PCR) is frequently used for gene expression quantification, at the transcriptional level, due to its reproducibility and sensitivity. The method has also become the preferred method for validating results obtained by other techniques, such as microarrays or deep sequencing.

Herein we present our data obtained using an innovative high throughput transcriptome profiling approach of human OR genes, in WHOM of much larger set of 26 individuals.

## Materials and Methods

### Ethics statement

This project was approved by the Erasme Hospital ethics committee (ULB, Brussels, Belgium: P2011/135 and A2013/050).

### Patients and tissues specimens

WHOM were collected 27±12 hours post-mortem from 26 individuals (13 women and 13 men; aged from 39 to 81 years, with an average of 67±11 years for women and 63±12 years for men). Most individuals were of European origin. For each patient, the clinical information is summarized in Table 1. Patients with a history of dysosmia or rhinologic diseases, including allergic rhinitis and chronic sinusitis, were excluded. We also investigated the history of smoking, associated with smell's disorder probably related to alterations of the olfactory mucosa [51], [52]. Amongst the 26 subjects, 8 were smokers and the smoking status was unknown for 4 of them.

**Table 1:** Patient's data.

Patient	Age (years)	Sex	RIN*	Smoking**	Cause of death	Delay***	Origin
1	46	F	7.7	−	Digestive hemorrhage	25	Africa
2	50	F	6.8	Φ	Cerebral hemorrhage	49	European
3	59	F	7.8	+	Septic shock	27	European
4	61	F	8.1	+	Cerebral hemorrhage	16	European
5	61	F	7.4	Φ	Cerebral hemorrhage	51	European
6	68	F	5.9	−	Septic shock	26	European
7	70	F	7.4	Φ	Cerebral hemorrhage	48	European
8	72	F	8.5	−	Aortic dissection	9	European
9	75	F	6.8	Δ	Cardiogenic shock	11	European
10	75	F	7.5	−	Digestive hemorrhage	23	European
11	79	F	6.3	−	Septic shock	21	European
12	79	F	7.5	−	Cardiac infarction	24	European
13	81	F	6.7	−	Cardiac infarction	23	European
14	39	M	7.2	−	Cardiopulmonary stop on hypoxemia	17	European
15	50	M	6.5	−	Cerebral hemorrhage	24	European
16	52	M	8.4	+	Septic shock	27	European
17	53	M	7.9	+	Cerebral hemorrhage	16	European
18	54	M	6.6	Δ	Cryptococcal meningitis	22	European
19	65	M	7.7	−	Respiratory failure	43	European
20	67	M	6.5	+	Septic shock	24	European
21	69	M	7	+	Respiratory failure	48	European
22	70	M	7.3	−	Digestive hemorrhage	25	European
23	74	M	6.8	Φ	Aortic dissection	46	European
24	74	M	9	+	Septic shock lung	14	European
25	75	M	6.4	−	Septic Shock	19	European
26	79	M	6.8	+	Cardiac infarction	30	European

*RNA integrity number.

**Smoking: −, never; Δ, past; +, current; Φ, unknow.

***Delay in hours between death and sample collection.

The WHOM was accurately dissected from the septum, the cribriform plate, the middle and the superior turbinates. As the boundaries between the olfactory and the respiratory epithelium are not clearly defined in humans [53], the septal mucosa was dissected up to the lower limit of the middle turbinate. A control tissue was taken from the mucosa of the inferior turbinate. The dissected tissue samples (about 3.5×5 cm from each side of the olfactory cleft mucosa) were collected, frozen in liquid nitrogen and stored immediately at −80°C.

### Total RNA isolation

Frozen WHOM was crushed in liquid nitrogen. Total RNA was purified and treated with DNase using the RNeasy kit (Qiagen) according to manufacturer instructions. DNase treatment is mandatory as intron spanning primers is not possible due to lack of introns in the OR genes. Quantitative and qualitative assessment of RNA samples (pooled from each side) was performed by NanoDrop spectophotometry (Thermo Scientific) and by microfluidic analysis using a 2100 Bioanalyser (Agilent Technologies). This latter technique produces an electropherogram allowing the evaluation of the integrity of the 18S and 28S ribosomal RNAs (Figure S1A and S1B) and the algorithm assigns a RNA integrity number (RIN) ranging from 1 to 10, where 10 corresponds to ideally intact RNA and 1 to highly degraded RNA (Table 1).

### cDNA synthesis

Total RNA (1 µg per sample-loading port of the 48 PCR reaction channels) was used in the reverse transcription (RT) Quantiscript reaction (Qiagen), performed with a combination of oligo-dT primer and random hexamers following the manufacturer's protocol. Each RNA sample was additionally run on one port (feeding 48 PCR assays) of the TaqMan Low Density Array (TLDA) in the absence of reverse transcriptase (RT-) to assess its potential contamination by genomic DNA. The latter, resulted for all samples, in a borderline amplification for a small subset of the large panel of intronless genes tested. On average, 90% of the PCR yielded a quantification cycle (C_q_) value labeled as undetermined or above 35 cycles. The remaining 10% gave an average C_q_ of 34.1±1.1 indicating a potential low residual genomic DNA contamination.

### TLDA design and preparation

A customized TLDA was designed in collaboration with Applied Biosystems. The design process for the assays is described in the White Paper TaqMan Gene Expression Assays from Applied Biosystems. The software TaqExpress was used for the design. Whenever possible, the assays were designed to amplify part of the gene coding sequence. The context sequence determines approximate assay position and the assay IDs allows retrieving the details from Applied Biosystems website (Table S1).

The 384 wells of the TLDA contain FAM dye-labeled NFQ probes and primers for an internal control (GAPDH, 4 wells), 10 endogenous control genes exhibiting low differential expression across tissues (MRPL19, CASC3, POLR2A, CDKN1B, TBP, RPL30, PSMC4, YWHAZ, UBC, PPIA), 356 human OR genes, and 6 reference genes specifically expressed in olfactory epithelium (CNGA2, GNAL, ADCY3, RIC8B, RTP1, OBP2A&2B) [50], [54]. cDNA (pre-mixed with TaqMan Universal PCR Master Mix) was loaded onto the TLDA and PCR amplifications were performed in a 7900HT Thermocycler (Applied Biosystems). Thermal cycling conditions used were: 2 min at 50°C, 10 min at 94.5°C, followed by 40 cycles at 97°C for 30 sec, and 59.7°C for 1 min.

### Real-time PCR with genomic DNA

One TLDA card was run with 150 ng (per port) of a pooled human genomic DNA from Clontech to evaluate the efficacy of the assays.

### Real-time PCR with plasmid DNA

One TLDA card was run with 30 pg (per port) of a pool of 30 OR coding plasmids cloned by ChemCom to evaluate the specificity of the assays. The receptors chosen to perform this experiment were spread throughout the different families of OR genes represented by an unrooted tree based on similarity of amino acid properties. One pg of each plasmid represents about 3000 molecules of specific plasmid per PCR.

### TLDA analysis and Statistical analysis

The real-time PCR focuses on the exponential phase, where amplification doubles target templates, following the exponential amplification (2^n^ where n is the number of cycles). The real-time PCR instrument calculates a C_q_ value representing the PCR cycle at which the reaction reaches a fluorescent intensity threshold above background. For C_q_ calculation, the threshold was manually set at ΔRn = 0.1 for all samples and all targets (threshold set within the 2^n^ exponential amplification phase). The results were analyzed using the Sequence Detection Systems (SDS) version 2.4 and Qbase^+^ software packages [55]. Determination of the optimal number of reference genes for the normalization of qPCR data was performed using the geNorm algorithm [56].

Association analyses were performed with R 2.14.1 [R Development Core Team (2008). R: A language and environment for statistical computing. R Foundation for Statistical Computing, Vienna, Austria. ISBN 3-900051-07-0, URL http://www.R-project.org.]. Significance Analysis of Microarray (SAM) [http://www.pnas.org.gate1.inist.fr/content/98/9/5116.full] was performed using the samr package v2.0 Genes with q-value below 0.05 were considered significant. For each variable (i.e. age, sex and smoking status) SAM was performed to find the receptors presenting expressions individually associated with each variable. To assess whether the expressions of all receptors were globally associated with one of these variables, the sum of the square of scores of association (Pearson correlation coefficients for age, t-scores for the two other variables) of all the receptors expressions was compared to a null-distribution of the sum of the scores of associations obtained after 10.000 permutations of the patient labels. Heatmap visualization was obtained with the heatmap.2 function within the gplots v2.10.1 package [gplots: Various R programming tools for plotting data (2011), Gregory R. Warnes, URL http://CRAN.R-project.org/package=gplots].

## Results

A 384-customized TLDA was designed to investigate the gene expression of a large array of OR genes from 26 WHOM samples. All experiments were performed according to the MIQE (minimum information for publication of quantitative real-time PCR experiments) guidelines [57].

### Analysis of RNA purity and integrity

The 260/280 and 260/230 OD ratios were measured for all RNA samples to assess respectively the purity of RNA with respect to protein contamination and residual organic solvent. All samples used showed a 260/280 and 260/230 OD ratios between 1.8 and 2.0, indicative of good quality RNA with minimal contaminations. RNA integrity was also assessed, and samples characterized by RIN (RNA integrity number) ranging from 5.9 to 9.0 were used, the average ±SD being 7.3±0.8 (Figure S1A, S1B, Table 1). These RIN values are usually considered acceptable for qRT-PCR experiments [58]. No correlation between the RIN and the delay between death and sample collection was observed (Statistical analysis reveal a p value = 0.36 for a Pearson's correlation with a R^2^ = 0.035).

### Selection of candidate reference genes

Classical endogenous control genes, exhibiting minimal differential expression across different tissues (MRPL19, CASC3, POLR2A, CDKN1B, TBP, RPL30, PSMC4, YWHAZ, UBC, PPIA) were added to the TLDA, in order to perform a first technical normalization and to compare expression of genes from different tissues as for example WHOM and inferior turbinate. The structure of WHOM is often patchy and contains a significant proportion of respiratory epithelium [54], [59]. Therefore, to compare expression of genes from different WHOM, assays for tissue specific olfactory epithelium reference genes were also added to the TLDA for a second biological normalization purpose. The six selected genes were CNGA2, GNAL, ADCY3, RIC8B, RTP1 and OBP2A&2B.

### Expression profiling and stability analysis of candidate reference genes

**Figure 1:**
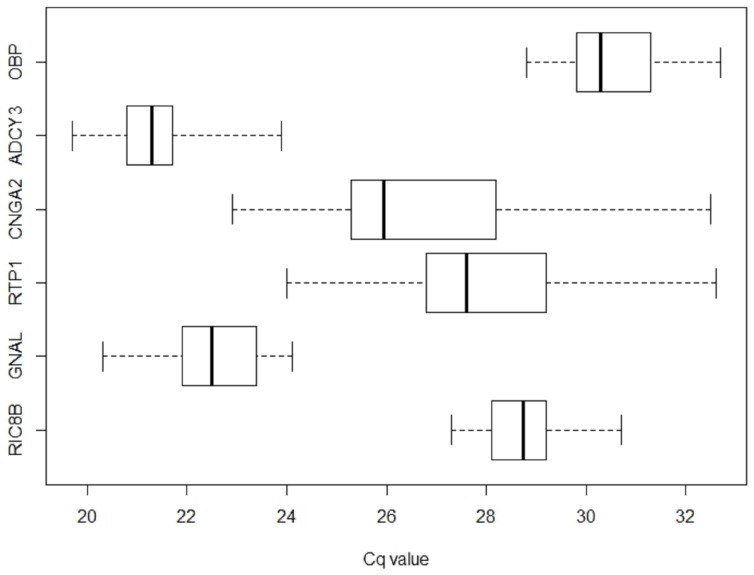
Expression profiling of candidate reference genes in whole human olfactory mucosa. Box plot graph on C_q_ obtained for the reference genes specific for the olfactory epithelium across the 26 individuals samples. Left and right box limits are first and third quartiles. The inner line conventionally marks the median. Whiskers show the extreme of the series.

Average C_q_ values of classical endogenous reference genes was 17.1±0.7 for UBC (mean ± SD), 17.6±0.5 for GAPDH, 18.0±0.6 for PPIA, 20.0±0.6 for CDKN1B, 20.4±0.6 for CASC3, 21.5±0.7 for PSMC4, 21.7±0.6 for POLR2A, 22.1±0.8 for YWHAZ, 22.6±0.9 for RPL30, 22.8±3.6 for MRPL19 and 24±0.6 for TBP (Table S1). Their stabilities were evaluated by the geNorm algorithm [56] and the geometric mean of 3 stably expressed classical endogenous reference genes (CASC3, PSMC4, CDKN1B) were selected to technically normalize the results. C_q_ of the olfactory epithelium-specific reference genes (Ric8B, GNAL, RTP1, CNGA2, ADCY3, OBP) are shown in the box plots of Figure 1. The distribution of the olfactory epithelium-specific reference genes C_q_ provides a global representation of the variation of reference gene expression as well as information on their relative abundance. More highly expressed genes are associated with lower C_q_. Average C_q_ values ranged from 21.3±0.8 (ADCY3) to 30.6±1.1 (OBP, means ±SD, n = 26) (Table S1). As suggested in Khan et al. [54], in view of obtaining a biologically relevant normalization, the geometric mean of the six specific reference genes provided a normalizing factor, rather than a factor from a single reference gene.

### Real-time PCR with genomic DNA

One TLDA card was run with a pool of human genomic DNA to evaluate the assays (individual gene efficiency amplification). The C_q_ average value of 351 detected OR genes is 24.5±0.8 (Table S2) suggesting similar amplification rates for all the genes, as expected as the number of targets is identical for all genes in a human genome. Furthermore 21 assays gave an expected undetermined C_q_ values because they correspond either to reference genes or to 4 OR genes for which the assays are designed with intron spanning primers. These assays are identified by a suffix ‘_m’ in the assayID. One out of 4 GAPDH assays gave a non-expected value of 36 and PPIA gave a non-expected value of 27 whereas these assays are designed with intron spanning primers. This reflects a slight non-significant amplification compared with results obtained on RNA (delta-C_q_ of 18 for GAPDH and 9 for PPIA). Finally, one target (OR2A14) showed an abnormal C_q_ value of 12.4, therefore this assays has not been taken into account for the qRT-PCR analysis.

### Real-time PCR with plasmid DNA

One TLDA card was run with a pool of 30 OR coding plasmids to evaluate the specificity of the assays. The expected specific PCR amplification of the 30 targets gives an average C_q_ value of 25.4±1.1, (mean ±SD, n = 30). However, we observe a non-specific amplification for 15 additional receptors. For 11 of them, the average C_q_ value is 32.5±1.8, (mean ±SD, n = 11) which reflects a delta-C_q_ value of 7 as compared to specific amplification. In this case, the non-specific amplification is then negligible. For 4 of them, the average C_q_ value is 25.0±0.8, (mean ±SD, n = 4) which reflects no difference as compared to specific amplification. These 4 couples of genes OR2L3 and OR2L8 (97% identity on the entire DNA sequence), OR52E6 and OR52E8 (90% identity), OR52I1 and OR52I2 (97% identity) and OR5D16 and OR5D18 (83% identity) cannot be discriminated by this TLDA card. Then for the 30 OR coding plasmids, 88% of the PCR amplifications are specific for the target.

### Inter-run calibration

**Figure 2:**
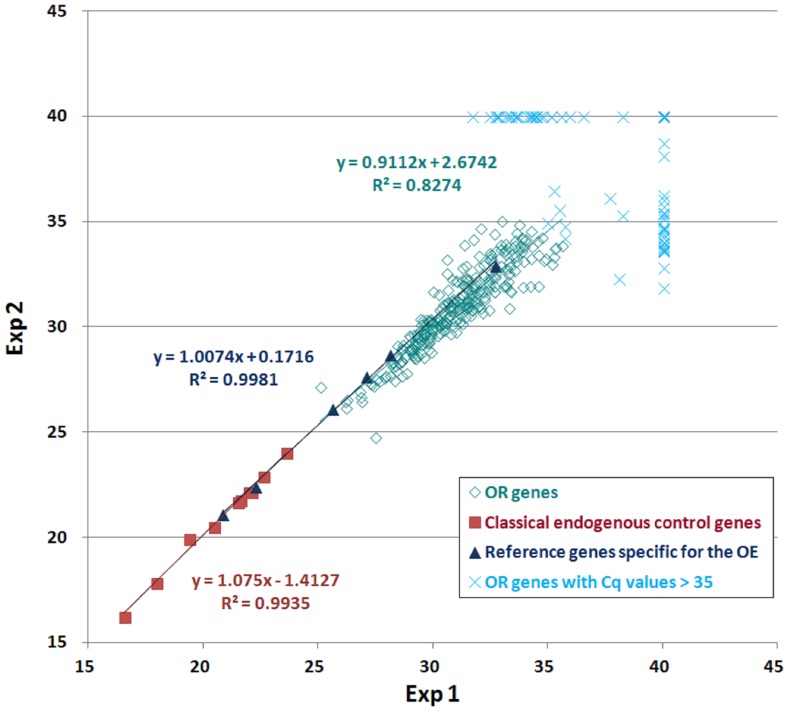
Inter-run calibration analysis. Plotted expression pattern correlation for all detected targets (C_q_ average below 35) from the same RNA WHOM sample in two different runs. OR genes (green **◊**), reference genes specific for the olfactory epithelium (blue **Δ**) and classical endogenous control genes (red ▪). C_q_ values above 35 are not reproducible (turquoise **x**). R^2^ is the coefficient of correlation.

Three different experiments were conducted to run the 26 samples, to correct for possible run-to-run variation whenever all samples are not analyzed in the same run, identical sample have been tested in all runs. Figure 2 shows the correlation between C_q_ values for all detected targets (ie. C_q_ average <35) from the same ARN sample in two different runs. C_q_ values above 35 are not reliable because duplicates are not reproducible. The correlation coefficient reaches 0.83 and the intercept is 0.91 for the detected OR genes. The correlation coefficient reaches 0.99 for the olfactory epithelium-specific reference genes and for classical endogenous control genes.

### Expression profiling of olfactory receptors genes in WHOM

On average, for the 26 samples, C_q_ values computed from amplification plots of 355 OR genes range between 25.8 and 39.8 (Table S1). These results reflect a low expression of the OR genes compared to other genes involved in the olfactory cascade. One target (OR2A14) shows an abnormal amplification plot with a C_q_ value of 16.6±7.3 (mean ±SD); this assays will not be taken into account for the analysis. On average, 62±29 (mean ±SD) OR genes per sample gave an undetermined C_q_ value which was arbitrarily assigned to 40 cycles to allow the calculation of an average C_q_ values. 74±34 (mean ±SD) OR genes per sample gave a C_q_ value above 35 were considered as expressed at very low level or not expressed at all.

To make a more quantitative analysis, the C_q_ values of each OR were converted into normalized relative quantities (NRQ) following the method previously described [55]. Briefly, we apply the delta-C_q_ quantification model using the average C_q_ obtained for all ORs in the 26 individuals as calibrator (here 32.7) which is transformed into relative quantities using the exponential function, so results are fully equivalent and thus only rescaled. Then the normalization of relative quantities was performed with the geometric mean of the multiple stably expressed classical endogenous reference genes (CASC3, PSMC4, CDKN1B) defined by the geNorm algorithm [56] and followed by the normalization with the geometric mean of the six reference genes specific for olfactory epithelium. Results obtained on genomic DNA and on specific OR coding plasmids allowed to calculate the approximate number of copies of target for 20 ng RNA engaged in the RT-PCR reaction (Table S3).

**Figure 3:**
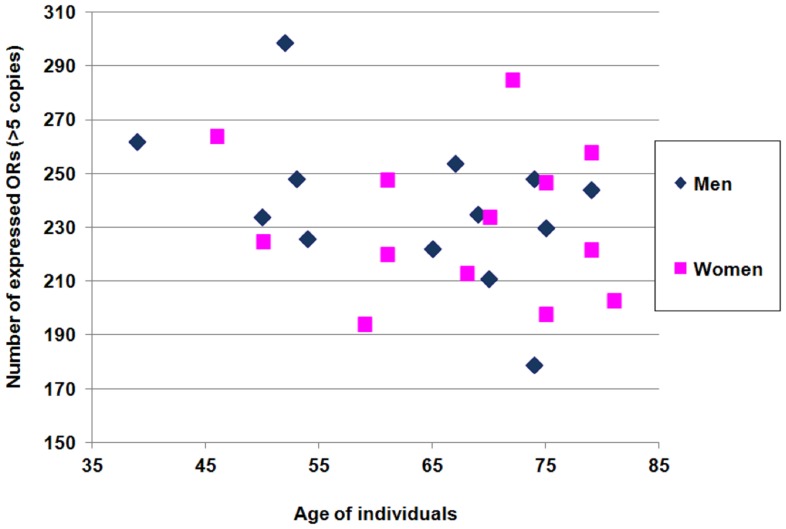
Number of OR genes expressed for each individual in function of age. Scatterplot of the number of OR genes expressed at a level above 5 copies/20 ng RNA for each individuals. Women are colored in pink and men in blue.

For each individual, the detected OR gene number (>5 copies/20 ng RNA) was counted (Figure 3). This cut-off value corresponds to a C_q_ value ≥35.3. On average, on the 355 OR gene targets, the detected OR gene number is 232±28 for women and 238±28 for men.

**Figure 4:**
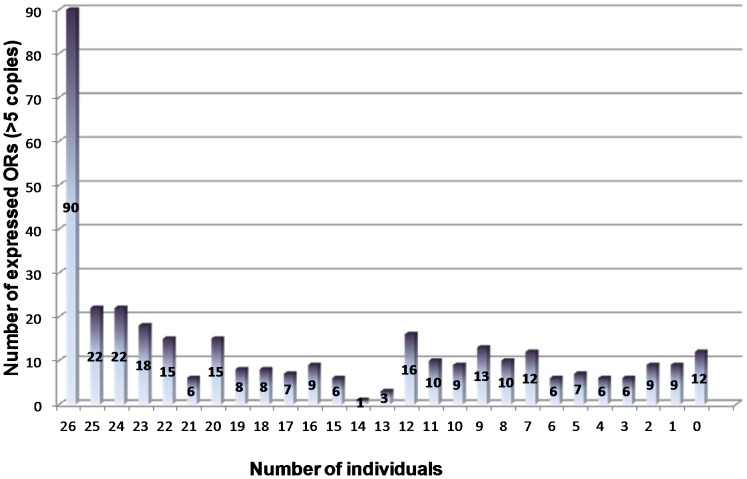
Expression frequency of OR genes in the population of 26 individuals. The bar chart represents the number of expressed OR genes (>5 copies/20 ng RNA) as a function of the number of expressing individuals, e.g. the number of expressed OR genes in all tested individuals (26) corresponds to 90.

There is a substantial difference in the expressed OR gene repertoire of each of the samples. A set of 90 human OR genes were detected (>5 copies/20 ng RNA) in all tested individuals. Another set of 140 human OR genes were detected not in all tested individuals but in more than half of the population (in 13 individuals and more on 26) and a third set composed of 125 human OR genes were more rarely detected (in less than 13 individuals on 26) (Figure 4).

**Figure 5:**
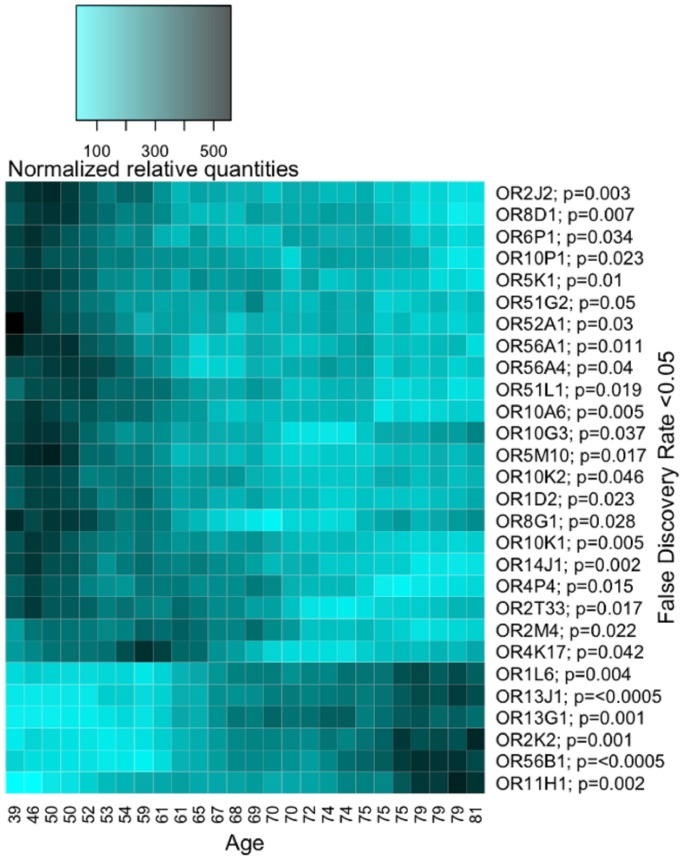
The expression of several OR genes is statistically related with age. 22 OR genes showed a decreased expression profile and 7 OR genes showed an increased expression profile. The False discovery rate (FDR) calculated by SAM is <0.05 for all represented genes and the p value of the Spearman's correlation is <0.05 are indicated next to the name of the ORs in the heatmap.

Globally, the OR gene expression was not associated with age (p value = 0.19), sex (p value = 0.23) or smoking (p value = 0.66, Pearson's correlation). Individually, 22 OR genes showed a decreased profile and 7 OR genes showed an increased profile related with age (Figure 5). There is no significant association between individual OR gene expression and sex or smoking.

**Figure 6:**
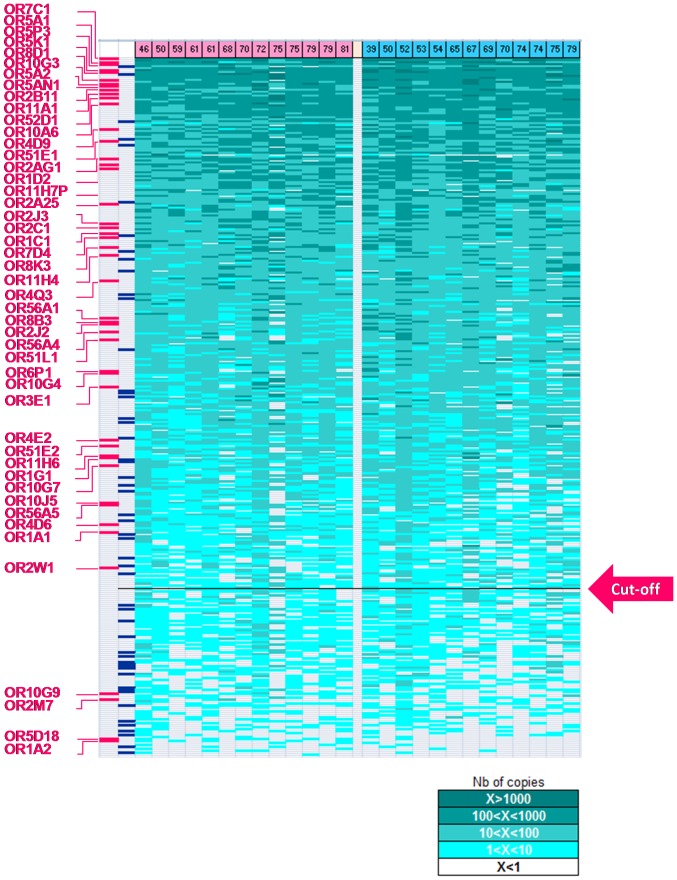
Expression profiling of OR genes in whole human olfactory mucosa. For each of the 355 OR genes (rows), the RNA copies number were estimated from normalized relative quantities obtained for each of the 26 individuals (columns). OR genes have been ranked according to their expression, from higher to lower. RNA copies number obtained for each individual are also indicated according to the green color code to show the good consistency of the inter-individual expression. OR genes with an average copies number below a cut-off of 5 copies/20 ng RNA (red arrow; right) are considered as to low or non-expressed. Age of the individuals are shown above the figure, women are colored in pink and men in blue. Published deorphanized receptors are highlighted in red on the left. Potentially non-functional OR genes are highlighted in blue on the left as well.

Figure 6 shows OR genes ranked in function of their expression level, from the highest to lowest. It shows 273 (77%) human OR genes above the cut-off value of 5 copies/20 ng RNA. No significant enrichment in class I or class II ORs is observed in the expressed set. Indeed, 17.6% of OR genes expressed belong to class I while 15.2% of the OR genes tested belong to this class.

Interestingly, most of the published deorphanized olfactory receptors [5]–[11], [13]–[20] are found into the set of expressed OR genes (Figure 6). Indeed, we count 43 expressed OR genes among the 47 deorphanized receptors described in the literature which are tested in this study. In other words, 16% of expressed OR genes are deorphanized while this percentage drops down to 4.8% for non-expressed OR genes (p value = 0.009, Fisher's exact test).

An inverse distribution is observed with potentially non-functional OR genes. Expression levels of 52 OR genes with mutations affecting positions in the consensus amino acid motifs specific for OR genes [60] were analyzed. These receptors, although regarded as intact OR genes, harbor a mutation affecting P or Y in the LHT**P**M**Y** motif, or affecting M, R or the second A in the **M**AYD**R**YV**A**IC motif, or Y in the S**Y** motif or finally, on H in the FSTCSS**H** motif. All known variants of these OR genes, correspond to a mutated haplotype of these highly conserved positions [48]. Presumably, these receptors are no longer functional (highlighted in blue in the Figure 6). We observed that 25% of non-expressed OR genes are potentially non-functional whereas 11.3% from the expressed set are potentially non-functional (p value = 0.002, Fisher's exact test). In addition, the average RNA copy numbers of the 47 deorphanized receptors (174±247) and of the 52 potentially non-functional ORs (48±115) are significantly different (p value = 0.002, Student's t-Test, two-tailed distribution, two-samples with unequal variance).

As shown in Table 2, the average RNA copy number varies drastically among the 47 reported deorphanized receptors. The most expressed OR gene corresponds to OR7C1 with an estimate average copy number of about 1108/20 ng RNA. A huge difference has also been noted in the expression of the four closely related paralogs, OR10G3, OR10G4, OR10G7 and OR10G9 that respond to ethyl vanillin and eugenol [5]. Indeed OR10G3 is well expressed in 25/26 WHOM samples with an average of 487 copies/20 ng RNA. OR10G4 and OR10G7 are moderately expressed (with an average of 29 and 13 copies/20 ng RNA respectively) and OR10G9 is detected only in 3 WHOM samples above the cut-off of 5 copies (with an average of 2 copies/20 ng RNA). We can count 19 ORs expressed by the 26 individuals among the 47 genes. In others words, 21% of the group of 90 receptors expressed in every individual were deorphanized. The 4 non-expressed ORs (OR1A2, OR2M7, OR5D18, OR10G9) are expressed only in 1, 4, 0 and 3 individuals respectively.

**Table 2:** RNA copies number average and number of individuals that express reported deorphanized olfactory receptors.

OR name	Agonist	Reference	Copies number average (per 20 ng RNA)	Number of individuals that express
OR1A1	Dihydrojasmone	[15]	7±8	12
OR1A2	Citronellal	[17]	0.6±1	1
OR1C1	Linalool	[5]	77±64	26
OR1D2	Bourgeonal	[19]	146±117	26
OR1G1	1-nonanol	[16]	14±48	11
OR2A25	Geranyl acetate	[5]	103±95	26
OR2AG1	Amyl butyrate	[11]	157±94	26
OR2B11	Coumarin	[5]	365±615	26
OR2C1	Octanethiol	[15]	78±71	26
OR2J2	1-octanol	[15]	37±46	23
OR2J3	Cis-3-hexen-1-ol	[13]	82±118	25
OR2M7	Citronellol	[15]	2±4	4
OR2W1	1-octanol	[15]	6±13	7
OR3A1	Helional	[20]	26±33	23
OR4D6	β-ionone	[8]	8±13	11
OR4D9	β-ionone	[8]	190±216	26
OR4E2	Amyl acetate	[10]	17±15	20
OR4Q3	Eugenol	[10]	50±50	22
OR5A1	β-ionone	[8]	737±1018	25
OR5A2	β-ionone	[8]	435±386	26
OR5AN1	Muscone	[18]	377±290	26
OR5D18	Eugenol	[6]	0.7±1	0
OR5K1	Eugenol methyl ether	[5]	598±412	26
OR5P3	(+)-carvone	[15]	752±642	26
OR6P1	Anisaldehyde	[10]	29±59	16
OR7C1	Androstadienone	[10]	1108±849	26
OR7D4	Androstenone	[9]	74±98	24
OR8B3	(+)-carvone	[10]	37±50	22
OR8D1	4,5-dimethyl-3-hydroxy-2,5-dihydrofuran-2-one	[5]	558±572	25
OR8K3	(+)-menthol	[5]	66±80	21
OR10A6	3-phenyl propyl propionate	[10]	286±273	26
OR10G3	Ethyl vanillin	[5]	487±443	25
OR10G4	Ethyl vanillin	[5]	29±35	22
OR10G7	Eugenol	[5]	13±12	19
OR10G9	Ethyl vanillin	[5]	2±3	3
OR10J5	Lyral	[15]	10±9	19
OR11A1	2-ethyl fenchol	[5]	324±266	26
OR11H4	Isovaleric acid	[14]	64±51	26
OR11H6	Isovaleric acid	[14]	15±12	22
OR11H7P	Isovaleric acid	[14]	142±100	26
OR51E1	Nonanoic acid	[7]	170±266	26
OR51E2	Propionic acid	[15]	16±20	20
OR51L1	4-allylphenylacetate	[15]	35±33	22
OR52D1	Ethyl isobutyrate	[16]	317±272	26
OR56A1	Decyl aldehyde	[5]	39±41	26
OR56A4	Decyl aldehyde	[5]	37±39	25
OR56A5	Decyl aldehyde	[5]	9±11	15

### Expression profiling of olfactory receptor genes in inferior turbinate sample

**Figure 7:**
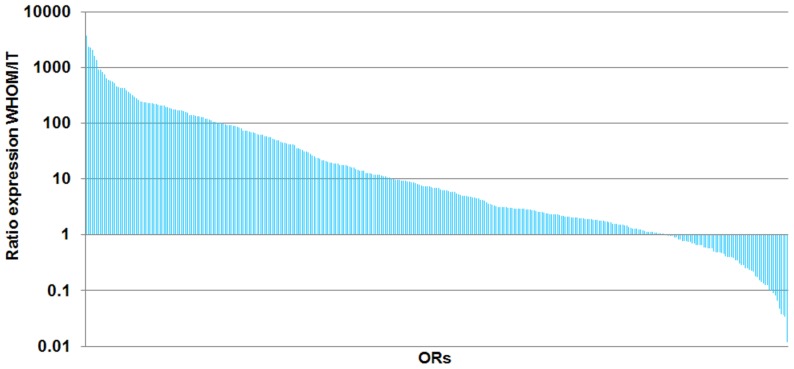
Ratio between normalized relative quantities of RNA obtained for olfactory mucosa and for inferior turbinate. For each of the 355 OR genes, the ratio is calculated from the mean of normalized relative quantities obtained for the 26 individuals for whole human olfactory mucosa (WHOM) and from the normalized relative quantities obtained for inferior turbinate (IT; n = 1).

One sample of inferior turbinate (IT) has been tested in a TLDA and gives 210 undetermined C_q_ values compared to 62 on average in WHOM tissues. This observation reflects the non-detection of OR gene expression, expected for a non-olfactory tissue. To make a more quantitative analysis, the C_q_ values were converted into normalized relative quantities with classical endogenous stably expressed reference genes (CASC3, PSMC4, CDKN1B) defined by the geNorm algorithm. Figure 7 shows results obtained for OR gene expression ratio between WHOM and inferior turbinate. We observe 250 OR genes (70%) more expressed in WHOM than in inferior turbinate (ratio WHOM/IT≥2) (Table S4). Some reference genes specific for olfactory sensory neurons (CNGA2 and Ric8B) are significantly expressed more in WHOM than in IT; RTP1 and ADCY3 are expressed a little more in WHOM than in IT while OBP and GNAL are detected in equivalent amount in both tissues.

## Discussion

Although the OR gene family was discovered over 20 years ago by Buck and Axel, few data are available on their expression in human olfactory mucosa, contrasting with the recent significant increase of results on the genetic polymorphism of OR genes [48]. This probably reflects the difficulty to acquire human tissue and obtain good quality RNA from WHOM.

We report here the first extensive high throughput transcriptome profiling of OR gene expression directly by real-time reverse transcription PCR performed furthermore on WHOM from a relatively large population of 26 patients. Indeed, our study was focused on the expression of 356 predicted functional OR genes among the 851 OR loci scattered throughout the human genome.

Only a small percentage of the olfactory mucosa consists of olfactory sensory neurons. Moreover, the boundaries of the WHOM are unclear and the tissue is sometimes replaced by respiratory epithelium. Furthermore, the fraction of olfactory sensory neurons can vary significantly from one sample to another. Therefore, to allow OR gene expression comparisons between individual WHOM samples, normalization is mandatory. Consequently, we therefore normalized our expression data from the individual WHOM, with 6 so called tissue specific reference genes, that are expressed specifically by olfactory sensory neurons as previously described by Khan et al. [54].

Our results show that 77% of human intact OR gene repertoire are expressed with an average level above 5 copies/20 ng RNA. A set of 90 human OR messengers were detected in all tested individuals. In addition, 70% of the human OR gene repertoire were found more expressed in WHOM than in the inferior turbinate (ratio WHOM/IT ≥2). Along with the widespread genetic variation reported for human OR protein coding regions, that correlates to individual differences in odorous perception [8], [9], [13], [14] and with genetic variations in auxiliary olfactory genes [50], this differential expression of ORs in the WHOM could establish the basis to a well-documented inter-individual variation in olfactory sensitivity.

Statistical analysis was performed for each clinical variable (i.e. age, sex and smoking status) to assess if receptor expressions were globally or individually associated. Our results indicate that OR gene expression is globally not associated with age, sex or smoking status. However, we are not able to detect if these clinical factors may reduce the absolute amount of olfactory sensory neurons. As we have normalized our data with specific references genes of olfactory sensory neurons, we have not taken into account the absolute amount of olfactory tissue. Therefore, the OR gene expression is described relatively to olfactory sensory neurons. Furthermore, at this point, our results cannot explain the decrease of olfactory performance related to age or smoking [51], [52], [61].

Nevertheless, our results show that individually, the expression of 22 OR genes seems to decrease significantly with age, and the expression of 7 OR genes seems to increase significantly. These results can be compared to those of Khan et al. [54] where the majority of OR gene expression (58.4%) in mice remained stable during aging, while 32.8% presented downward profiles, 7.2% upward profiles and 1.7% of convex or concave profiles. We found no correlation between individual OR gene expression and sex or smoking, although some clinical observations show that these two conditions may influence smell abilities. These differences in smell abilities may occur at another level of the olfactory system than the OR gene expression.

Prior to the present work, only two studies had focused on the expression of the human OR gene family. In these studies, DNA microarray [45] or deep sequencing [50] were used as experimental approaches. Latter report focused on accessory proteins and presented results in the supplementary data only for one human olfactory epithelium biopsy. Over the 261 intact ORs overlapping with our study, 174 OR genes were found to be expressed in this olfactory epithelium biopsy (threshold set at a FPKM≥0.1) whereas we found 202 OR genes expressed in the WHOM (threshold set at a number of copies ≥5). 145 genes (i.e.72% of our expressed OR gene set) turned out to be common to both studies and 30 ORs are not expressed in WHOM according to both studies (Table S5). Therefore, our approach is in agreement with the previous study by Keydar et al. concerning expressed OR genes (p value = 0.0012, hypergeometric test) and for the expression levels of each OR genes (p value<0.001, Spearman's correlation) [50]. With respect to the Zhang et al. study [45], there are 319 intact ORs overlapping with our study. 202 OR genes were found to be more expressed in human olfactory epithelium than in other tissues by Zhang et al. (threshold set at a p value<0.01) whereas we found 225 OR genes preferentially expressed in WHOM (threshold set at a ratio WHOM/IT≥2). Furthermore, 142 genes (i.e. 63% of our expressed OR gene set) turned out to be common to both studies and 34 ORs are not expressed in WHOM according to both studies (Table S6). The overlap of 63% is exactly what would be expected if the two datasets are completely random with respect to each other (p value = 0.3, hypergeometric test). Moreover, no correlation between the expression levels of each OR genes could be observed between the two studies (p value = 0.96, Spearman's correlation). It is noteworthy that the non-olfactory tissues used to estimate a difference in gene expression are not the same. We compared the expression in the WHOM to the one of inferior turbinate, while human liver, lung, kidney, heart and testis were used in the study by Zhang et al. The latter therefore must be taken with caution, as non-olfactory expression of ORs have been reported for these different tissues, and could therefore bias the comparative results they obtained. We tried to exclude the set of non-olfactory expressed ORs from our comparison. Even excluding these receptors, we could not reveal a correlation between the current data and the Zhang et al. data. Another source of discrepancy between both studies relies on the proportion of the olfactory epithelium used to determine OR expression. In the publication by Zhang et al., it is not clear whether the analyzed tissues cover the entire olfactory mucosa or only a determined anatomical section. This difference might be highly significant as the distribution of olfactory receptors is not homogeneous in the olfactory epithelium of rodents [62], [63]. Importantly, though no data currently exist in humans. Another difficulty relies on the fact that the boundaries between the olfactory and the respiratory epithelium are not clearly defined in humans. Different publications report that the human olfactory epithelium is located on the nasal septum, the cribriform plate, the superior and the middle turbinate [53], [64], [65]. Consequently, this motivated us collecting all these anatomical regions. The mucosa of the nasal septum was dissected along the projection of the middle turbinate. Thus, our samples represent practically the totality of the olfactory mucosa. Finally, our results are in good agreement with those of Keydar et al., but not with Zhang et al. Moreover, we found no correlation between Keydar et al. and Zhang et al. (p value = 0.341, hypergeometric test and p value = 0.13, Spearman's correlation).

Interestingly, we observe an enrichment of functional deorphanized receptors in the set of expressed OR genes and an enrichment of potentially non-functional receptors into the set of non-expressed OR genes. This corroborates the observations of Zhang et al. The latter reports that 80% of intact OR genes and 67% of OR pseudogenes were found to be expressed in WHOM and moreover intact ORs appear to be expressed at a higher level on average than OR pseudogenes.

Taken together, these observations support the hypothesis predicting that if a gene is expressed, it is more likely to be functional. Indeed, a non-functional OR can lead to a defective targeting of olfactory sensory neurons in the olfactory bulb and therefore reduces the survival of these neurons [66]. More precisely, the proper targeting seems more related to OR-derived cAMP signals rather than the OR ability to bind an odorant [67]. The relation between OR genes functionality and expression could be further explored by studying the variants of expressed OR genes. Indeed, for known ORs already deorphanized, both functional and non-functional haplotypes have been described; consequently, it would be worth determining whether expressed allelic variants correspond preferentially to functional haplotypes.

A systematic study of OR gene expression profiles, expressed in non-olfactory tissues using deep sequencing analysis has been recently reported and provides a list of highly expressed OR genes [44]. From this list, 32 intact OR genes are common with our study. We confirmed that the majority of the non-olfactory tissues expressed OR genes (28/32; 87%) are also expressed in WHOM (more than 5 copies/20 ng RNA). 24 out of 32 (75%) non-olfactory tissues expressed OR genes are expressed more in WHOM than in inferior turbinate (ratio WHOM/IT ≥2). The remaining 8 OR genes are detected similarly in both tissues (0.96< ratio WHOM/IT1 <2). From this comparison, it does not seem that non-olfactory tissues expressed OR genes make up a separate group, with a putative non-olfactory function, that would clearly segregate from ORs expressed in the WHOM. However, for one particular receptor, OR2W1, we observed a very low expression level in the WHOM whereas it is well detected in pulmonary neuroendocrine cells [31]. Upon its deorphanization, this receptor was found have a broad spectrum of stimuli [15]. This OR is activated by more than 200 molecules and interacts with a large variety of chemical structures eliciting very different odors [Veithen et al., unpublished data]. Together, our results and those of Gu et al. [31] suggest a role for OR2W1 in the detection of volatile irritants in the human airways. Therefore, this receptor may offer an example of an OR family member that would actually not be only an olfactory mucosa odorant receptor.

Although our study represents the most extensive analysis of human OR expression in the olfactory mucosa, it does present some limitations. The considered population is relatively old. Indeed, because of the difficulty to obtain human material, samples from patients presenting characteristics that may affect the olfactory mucosa such as age and smoking, have not been discarded. However, these conditions do not appear to change the OR genes expression. On another hand, our study includes almost exclusively subjects of European origin and therefore does not explore the possible ethnic related variations of OR gene expression. Logically, therefore, we acknowledge that it would be worth extending the analysis to samples from other origins, if available.

Notwithstanding these points and since the majority of human olfactory receptors are not deorphanized, the information on the expression of OR genes in WHOM collected in this study offers an essential preliminary and lacking understanding that will allow focusing future research on frequently expressed and potentially functional olfactory receptors identified.

## Supporting Information

Figure S1 **Analysis of RNA integrity.** **A.** Electropherogram showing the integrity of 9 human olfactory epithelium RNA samples (lanes 1 to 9). **B.** Example of profile showing a RIN of 8.5 (sample 8, RNA from a woman of 72 years old) and the integrity of the 18S and 28S ribosomal RNA.
(TIF)

Table S1 **C_q_ values obtained for all OR genes and reference genes in all WHOM samples including assays ID from Applied Biosystems.**
(XLSX)

Table S2 **C_q_ values obtained for all OR genes and reference genes on genomic DNA and average C_q_ values obtained on WHOM RNA.**
(XLSX)

Table S3 **Copies number estimated for all OR genes and reference genes in all WHOM samples.**
(XLSX)

Table S4 **Ratio between normalized relative quantities obtained for WHOM and for inferior turbinate and the average copies number for all OR genes and reference genes.**
(XLSX)

Table S5 **Comparison between the 2 studies by Verbeurgt et al. and by Keydar et al.: listing of 145 ORs that are expressed in the WHOM according to both studies and listing of 30 ORs that are not expressed in the WHOM according to both studies.**
(XLSX)

Table S6 **Comparison between the 2 studies by Verbeurgt et al. and by Zhang et al.: listing of 142 ORs that are expressed in the WHOM according to both studies and listing of 34 ORs that are not expressed in the WHOM according to both studies.**
(XLSX)
