# Supplementary figures and images for: Profiling of Olfactory Receptor Gene Expression in Whole Human Olfactory Mucosa
Source: PLoS One. 2014 May 6;9(5):e96333. doi: 10.1371/journal.pone.0096333 (PMC4011832; doi:10.1371/journal.pone.0096333)

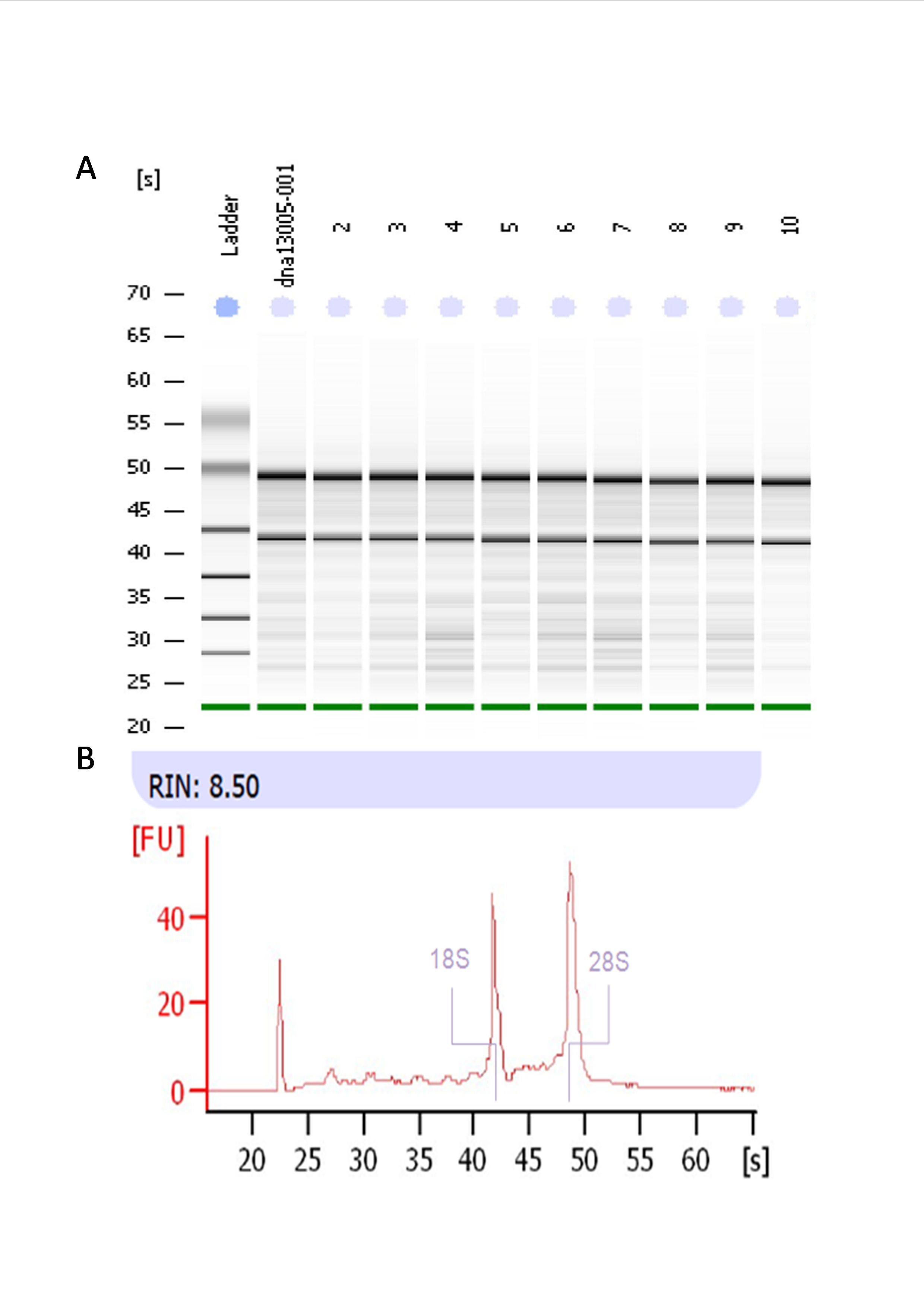

Supplement: Figure S1 — Analysis of RNA integrity. A. Electropherogram showing the integrity of 9 human olfactory epithelium RNA samples (lanes 1 to 9). B. Example of profile showing a RIN of 8.5 (sample 8, RNA from a woman of 72 years old) and the integrity of the 18S and 28S ribosomal RNA. (TIF) [file pone.0096333.s001.tif]
